# Outcome Differences between Conservatively Treated Acute Bony and Tendinous Mallet Fingers

**DOI:** 10.3390/jcm12206557

**Published:** 2023-10-16

**Authors:** Guy Rubin, Alaa Ammuri, Uri Diego Mano, Ravit Shay, Sigal Better Svorai, Ruty Sagiv, Nimrod Rozen

**Affiliations:** 1 Orthopedic Department, Emek Medical Center, Afula 1834111, Israel; 2 Rappaport Faculty of Medicine, Technion-Israel Institute of Technology, Haifa 3109601, Israel; 3 Occupational Therapy Unit, Emek Medical Center, Afula 1834111, Israel

**Keywords:** mallet finger, outcome, splint

## Abstract

Introduction: Tendinous and bony mallets are very different injuries that present with extensor lag at the distal interphalangeal joint. This study aimed to evaluate the differences in outcomes between acute bony and tendinous mallet fingers treated conservatively with splints. Materials and Methods: We retrospectively collected data on patients with acute tendinous or bony mallets who received conservative treatment in our occupational therapy clinic. The patients were examined at an outpatient clinic, where data on pain, extension lag, and loss of flexion were recorded. Outcomes were classified according to the criteria described by Crawford. Results: Data were collected from 133 patients (43 with bony and 90 with tendinous mallets). We found that bony mallet patients were predominantly younger (mean, 36 vs. 46 years), and more likely to be female (60% vs. 34%), than tendinous mallet patients. We also found that tendinous mallet injuries predominantly affected the middle and ring fingers, while bony mallet injuries predominantly affected the ring and little fingers. The initial extensor lag was worse in tendinous than in bony mallets (median, 28° vs. 15°). In addition, patients with bony mallets had significantly better outcomes with regard to the extension lag (median 0° vs. 5° *p* = 0.003) and the Crawford Criteria Assessment (*p* = 0.004), compared with those with tendinous mallets. Discussion: Mallet injuries, both tendinous and bony, are common. They are often studied together and typically treated in the same manner using extension splints. However, evidence clearly shows that these are different injuries which present in the same manner. This study reinforces these findings and suggests that the outcome of conservative treatment is better for bony than for tendinous mallet fingers.

## 1. Introduction

Mallet finger is a common injury, with an incidence rate of 10 per 100,000 population each year [1]. Mallet injuries can be classified into four types according to the Doyle system [2] (Table 1). Bony mallets are classified according to the Wehbé and Schneider classification [3] (Table 2).

Regardless of the mechanism of injury, mallet finger is treated by immobilizing the DIP joint in extension and maintaining that position for 6 to 8 weeks, followed by 2 to 4 weeks of night immobilization and with strenuous activity to prevent an extension. If the DIP joint is allowed to flex at any point in the initial 6 to 8 weeks, the integrity of the healing process is interrupted, and the immobilization regimen must start again. A gap of just 0.5 mm will lead to a 10° extensor lag [4]. Tendon repair is not a surgical option at this level of injury, although placement of a K-wire through the DIP joint is an option for some patients (in patients with chronic mallet, complex open injuries or patients who may be less compliant with an orthosis regimen). K-wire placement is also recommended if there is a fracture with disruption of the joint surface which is greater than 1/3 or if there is subluxation of the DIP joint. For most mallet finger injuries, the treatment of choice is immobilization with an orthosis. The PIP joint is left free in the orthosis [5].

A mallet finger treatment outcome assessment classification was proposed by Crawford [6]. It is the most commonly used classification for outcome assessment after mallet finger. An excellent outcome is no pain with full range of motion at the DIP joint, less than 10-degree extension deficit is a good outcome, 10–25 degrees of extension deficit with no pain is a fair outcome, and more than 25 degrees of extension deficit or persistent pain is considered a poor outcome.

Tendinous and bony mallets are very different injuries that present with extensor lag at the distal interphalangeal (DIP) joint. Tendinous mallets are often observed in older patients usually following low-energy injuries, and are often painless. Bony mallets occur at a younger age (mean, 40 years), are always high-energy injuries and are always painful [7,8,9,10]. Tendinous mallet injuries affect predominantly the middle and ring fingers while bony mallet injuries affect predominantly the ring and little fingers [3,11,12,13,14,15,16].

Bony mallet injuries are hyperextension injuries that should not be treated with hyperextension splints. Splints with a neutral or slight flexion are preferable. Sivakumar et al. recommended total immobilization of bony injuries for six weeks, whereas tendinous disruptions were usually splinted for at least eight weeks, to allow for the longer duration of tendon healing [17]. Giddins suggested that bony mallets needed only 3–4 weeks to heal, allowing earlier restoration of mobility [7].

This study aimed to evaluate the differences in outcomes between acute bony and tendinous mallet fingers following conservative treatment with splints. 

## 2. Materials and Methods

This retrospective study was approved by the local Institutional Review Board. We collected data on patients with acute tendinous (Doyle type 1) or bony (Doyle type 4 B,C/Wehbé, and Schneider type 1A,B) mallets that were treated conservatively in our occupational therapy clinic. Radiographs of the affected digit were used to distinguish between a bony and tendinous mallet injury. Lateral view radiograph was performed to identify the presence of an avulsion fracture at the DIPJ and to assess the degree of joint involvement and subluxation in bony mallet injuries. Patients under 18 years of age, those who underwent surgery, chronic cases (>4 weeks), and patients who did not follow the conservative protocol were excluded from the study of treatment outcomes.

There are many variations in the design of splints, but the principle is the same. All mallet finger splints are designed to maintain full extension or slight hyperextension at the DIP joint. It is critical that the orthosis fits well and is adjusted accordingly to ensure that an extensor lag does not develop, and proximal interphalangeal (PIP) range of motion is allowed. The splint should be used continuously and the DIP joint should be maintained in full extension even during skin hygiene care. Patients should be instructed on how to change the splint for periodic cleaning and examination of the skin without allowing the DIP joint to flex. Neglecting a mallet injury or incorrect treatment can lead to DIP joint dysfunction. A 1 mm lengthening of the terminal extensor tendon results in 25 degrees of extension lag, and a shortening of 1 mm will seriously restrict DIP joint flexion [18]. All patients in our study were treated by an occupational therapist and fitted with a custom-made circumferential thermoplastic orthosis. All orthotics were worn continuously for 6 weeks for the tendinous mallet and for 4 weeks for the bony mallet, followed by a 4-week graduated withdrawal and exercise program.

The patients were examined at an outpatient clinic after 6 months from the beginning of treatment, where data on pain, extension lag, and loss of flexion were recorded. Outcomes were classified according to the criteria described by Crawford [6] (Table 3).

## 3. Statistical Analysis

Data on demographic and clinical characteristics were collected and summarized in an Excel table as coded data. The means and standard deviations are used to report normally distributed continuous data. Non-normally distributed variables are expressed as medians (interquartile ranges). Categorical variables are reported as frequencies and percentages. Differences in continuous variables between the groups were assessed using the Student’s *t*-test for normally distributed data or the Mann–Whitney U test for non-normally distributed data. For categorical variables, differences were assessed using the χ^2^ test or Fisher’s exact test. Variables with ordered levels, for example the Crawford outcome criteria, were analyzed using the Cochran–Armitage test. Statistical analyses were performed using R software version 4.1.3. Statistical significance was set at *p* ≤ 0.05.

## 4. Results

A total of 133 patients underwent conservative treatment for mallet injuries (43 bony and 90 tendinous) between January 2016 and January 2023 (Table 4).

There was a statistically significant difference (*p* < 0.004) between the ages of patients with bony and tendinous mallets. The 43 patients with bony mallets had an average age of 36.23 (range, 13–83) years at the time of injury, and the 90 patients with tendinous mallets had an average age of 46.67 (range, 7–85) years at the time of injury. There was also a statistically significant difference (*p* = 0.005) between the sex ratios of the bony and tendinous mallet groups. The 43 patients with bony mallets included 17 (39.5%) men and 26 (60.5%) women, whereas the 90 patients with tendinous mallets included 59 (65.6%) men and 31 (34.4%) women.

There was no statistically significant difference in which hand was injured. There was a tendency for bony mallet injuries to involve the fourth and fifth fingers and tendinous mallet injuries to affect the third and fourth fingers, although this did not reach statistical significance (*p* = 0.057). Prior to treatment, the tendinous mallet group showed a greater extension lag than the bony mallet group, with median angles of 28° (*n* = 87) and 15° (*n* = 42), respectively (*p* < 0.001).

A total of 99 patients (21 with bony and 78 with tendinous mallets) met the inclusion criteria and were included in the outcome study (Table 5). There was a statistically significant difference (*p* = 0.004) between the bony and tendinous mallet extension lags after treatment. The bony mallet group had a median angle of 0°, whereas the tendinous mallet group had a median angle of 5°.

Crawford outcome criteria (Table 5) also revealed a statistically significant difference (*p* = 0.004) between the bony and tendinous mallet groups. The 21 bony mallet patients included 13 (61.9%) excellent, 4 (19%) good, and 4 (19%) fair results while the 78 tendinous mallet patients showed 9 (28.1%) excellent, 13 (40.6%) good, 5 (15.6%) fair, and 5 poor (15.6%) results.

## 5. Discussion

Mallet injuries, both tendinous and bony, are common. They are often studied together and typically treated in the same manner using extension splints. However, evidence clearly shows that these are different injuries which present in the same way [7]; the results of the present study support these findings. We found that patients with bony mallets were younger than those with tendinous mallets (mean, 36 vs. 46 years); however, patients in both groups of the present study were younger than those of a previous study (40 vs. 57 years) [7]. A male-to-female ratio of 60:40 for both injuries has been reported [7]; however, we found bony mallets to be more frequent in females (60:40). Clayton et al. found that patients were more likely to be male in the first three decades of life. The incidence was much lower in women in the second and third decades but rose steadily to match the incidence in men, with a peak in the sixth decade [1]. We also found that tendinous mallet injuries predominantly affected the middle and ring fingers, whereas bony mallet injuries predominantly affected the ring and little fingers.

We also examined the outcome differences between bony and tendinous mallets, finding that the bony mallet group had significantly better outcomes with regard to the extension lag and Crawford Criteria Assessment. Previous studies on bony mallets have reported favorable clinical outcomes. Goto et al. found a final extension lag of 8.5° and excellent or good results in 88% of patients, according to the criteria of Crawford [19]. Kalainov et al. found a final extension lag of 3° [20] and Thillemenn et al. found a final extension lag of 12° [21]. Tendinous mallets treated with orthosis also demonstrated good results, with a final extension lag of 5–16° [22,23,24,25,26]. We believe our findings are reflective of the superior healing capacity of bone fractures compared with tendons, and the smaller DIP joint extensor lag observed following bony compared with tendinous mallet injuries.

Giddins noticed that after 4 weeks the dorsal fracture fragment moves with the main fragment of the distal phalanx, that is, stability has been restored to the DIP joint. This suggests that extension splinting for bony mallet injuries need only last 4 weeks for some and perhaps all of these injuries which would fit with bone healing times in the hand. In contrast, the much slower extensor tendon healing time demands a longer period (6–8 weeks) of splintage for tendinous mallet injuries [7]. The recent work of Trickett and colleagues treating 218 bony mallet injuries with splintage suggests they “never” need surgery [16]. That would fit with bony mallet injuries being more stable than tendinous mallet injuries as shown by the much lesser extensor lag but does not explain the outcome for the rarer but recognized cases of distal phalanx proximal volar dislocation.

A limitation of this study is its retrospective design. A larger, prospective study is required to confirm these findings. Nevertheless, the results of the present study suggest that bony mallet injuries have better outcomes than tendinous mallet injuries, and that they therefore require a shorter splinting time of 4 weeks, compared with 6–8 weeks for slow-healing tendinous injuries.

## 6. Conclusions

This study compared the outcomes of patients treated conservatively for bony and tendinous mallet injuries, as although these are different injuries, they are often treated in the same way. This study shows that bony mallet injuries have better short-term outcomes than tendinous mallet injuries, with respect to both extensor lag and the Crawford outcome criteria.

## Figures and Tables

**Table 1:** Doyle classification of mallet fingers.

Type	Definition
1	Closed injury with or without small avulsion fracture
2	Open superficial soft tissue injury
3	Open deep soft tissue injury
4	Mallet fracture
4a	Growth plate fracture (pediatric)
4b	Fragment involves 20–50% of articular surface
4c	Fragment involves more than 50% of articular surface

**Table 2:** Wehbé and Schneider’s classification of bony mallet fingers.

Type	Definition
1	No DIP joint subluxation
2	DIP joint subluxation
3	Epiphyseal and physeal injuries
Subtype	
A	Avulsed fragment <1/3 of articular surface
B	Avulsed fragment 1/3–2/3 of articular surface
C	Avulsed fragment >2/3 of articular surface

DIP: distal interphalangeal.

**Table 3:** Crawford Criteria (1984) Assessment of Mallet Finger.

Grade	Characteristics of DIP Joint
Excellent	Full extension, Full flexion, No pain
Good	Extension deficit 0° to 10°, Full flexion, No pain
Fair	Extension deficit 10° to 25°, Any flexion loss, No pain
Poor	Extension deficit >25°, Persistent pain

DIP, distal interphalangeal.

**Table 4:** Patient demographics and injury characteristics.

Group	Bony Mallet (*n* = 43)	Tedinous (*n* = 90)	Total (*n* = 133)	*p* Value
Age				0.004 ^1^
N	43	90	133	
Mean (SD)	36.23 (17.91)	46.67 (19.91)	43.29 (19.83)	
Median	36.00	48.00	44.00	
Q1, Q3	22.00, 44.00	37.00, 61.75	27.00, 59.00	
Range	13.00–83.00	7.00–85.00	7.00–85.00	
Sex				0.005 ^2^
F	26 (60.5%)	31 (34.4%)	57 (42.9%)	
M	17 (39.5%)	59 (65.6%)	76 (57.1%)	
Side				0.439 ^2^
L	17 (39.5%)	42 (46.7%)	59 (44.4%)	
R	26 (60.5%)	48 (53.3%)	74 (55.6%)	
Finger				0.057 ^2^
N-Miss	1	2	3	
1	2 (4.8%)	1 (1.1%)	3 (2.3%)	
2	4 (9.5%)	6 (6.8%)	10 (7.7%)	
3	6 (14.3%)	31 (35.2%)	37 (28.5%)	
4	14 (33.3%)	31 (35.2%)	45 (34.6%)	
5	16 (38.1%)	19 (21.6%)	35 (26.9%)	
Angle before splint				< 0.001 ^1^
N	42	87	129	
N-Miss	1	3	4	
Mean (SD)	15.00 (10.20)	27.20 (11.49)	23.22 (12.45)	
Median	15.00	28.00	22.00	
Q1, Q3	8.50, 20.00	20.00, 33.00	15.00, 30.00	
Range	0.00–40.00	5.00–60.00	0.00–60.00	

^1^ Linear Model ANOVA. ^2^ Pearson’s Chi-squared test. Kruskal–Wallis rank sum test.

**Table 5:** Patient outcomes.

Group	Bony Mallet (*n* = 21)	Tendinous (*n* = 78)	Total (*n* = 99)	*p* Value
angle after splint				0.004 ^1^
N	21	68	89	
N-Miss	0	10	10	
Mean (SD)	1.90 (3.35)	8.19 (9.75)	6.71 (9.06)	
Median	0.00	5.00	4.00	
Q1, Q3	0.00, 5.00	0.00, 15.00	0.00, 10.00	
Range	0.00–10.00	0.00–35.00	0.00–35.00	
outcome_crawford				0.004 ^1^
N-Miss	0	46	46	
excellent	13 (61.9%)	9 (28.1%)	22 (41.5%)	
good	4 (19.0%)	13 (40.6%)	17 (32.1%)	
fair	4 (19.0%)	5 (15.6%)	9 (17.0%)	
poor	0 (0.0%)	5 (15.6%)	5 (9.4%)	

Data are presented as mean ±standard deviation or number (%). ^1^ Mann–Whitney U test.

## Data Availability

Not applicable.

## References

[1] Clayton R.A., Court-Brown C.M. (2008). The epidemiology of musculoskeletal tendinous and ligamentous injuries. Injury.

[2] Doyle J.R., Green D.P., Pederson C.W., Hotchkiss R.N. (1999). Extensor tendons: Acute injuries. Green’s Operative Hand Surgery.

[3] Wehbe M.A., Schneider L.H. (1984). Mallet fractures. J. Bone Jt. Surg. Am..

[4] Howell J.W., Peck F. (2013). Rehabilitation of flexor and extensor tendon injuries in the hand: Current updates. Injury.

[5] Cheung J.P., Fung B., Ip W.Y. (2012). Review on mallet finger treatment. Hand Surg..

[6] Crawford G.P. (1984). The molded polythene splint for mallet finger deformities. J. Hand Surg..

[7] Giddins G. (2022). Mallet Finger: Two Different Injuries. Hand Clin..

[8] Lange R.H., Engber W.D. (1983). Hyperextension mallet finger. Orthopedics.

[9] McMinn D.J. (1981). Mallet finger and fractures. Injury.

[10] Giddins G. (2021). Tendinous and bony mallet finger: Mechanisms of injury. J. Hand Surg. Eur. Vol..

[11] Pegoli L., Toh S., Arai K., Fukuda A., Nishikawa S., Vallejo I.G. (2003). The Ishiguro extension block technique for the treatment of mallet finger fracture: Indications and clinical results. J. Hand Surg..

[12] Stack H.G. (1969). Mallet Finger. Hand.

[13] Vernet P., Igeta Y., Facca S., Toader H., Hidalgo Diaz J.J., Liverneaux P. (2019). Treatment of tendinous mallet fingers using a Stack splint versus a dorsal glued splint. Eur. J. Orthop. Surg. Traumatol..

[14] Facca S., Nonnenmacher J., Liverneaux P. (2007). Treatment of mallet finger with dorsal nail glued splint: Retrospective analysis of 270 cases. Rev. Chir. Orthop. Reparatrice Appar. Mot..

[15] Moradi A., Braun Y., Oflazoglu K., Meijs T., Ring D., Chen N. (2017). Factors associated with subluxation in mallet fracture. J. Hand Surg. Eur. Vol..

[16] Trickett R.W., Brock J., Shewring D.J. (2021). The non-operative management of bony mallet injuries. J. Hand Surg. Eur. Vol..

[17] Sivakumar B.S., Graham D.J., Ledgard J.P., Lawson R.D. (2023). Acute Mallet Finger Injuries—A Review. J. Hand Surg..

[18] Schweitzer T.P., Rayan G.M. (2004). The terminal tendon of the digital extensor mechanism: Part II, kinematic study. J. Hand Surg..

[19] Goto K., Naito K., Nagura N., Sugiyama Y., Obata H., Kaneko A., Kawakita S., Kajihara H., Iwase Y., Ishijima M. (2021). Outcomes of conservative treatment for bony mallet fingers. Eur. J. Orthop. Surg. Traumatol..

[20] Kalainov D.M., Hoepfner P.E., Hartigan B.J., Carroll C., Genuario J. (2005). Nonsurgical treatment of closed mallet finger fractures. J. Hand Surg..

[21] Thillemann J.K., Thillemann T.M., Kristensen P.K., Foldager-Jensen A.D., Munk B. (2020). Splinting versus extension-block pinning of bony mallet finger: A randomized clinical trial. J. Hand Surg. Eur. Vol..

[22] Foucher G., Binhamer P., Cange S., Lenoble E. (1996). Long-term results of splintage for mallet finger. Int. Orthop..

[23] O’Brien L.J., Bailey M.J. (2011). Single blind, prospective, randomized controlled trial comparing dorsal aluminum and custom thermoplastic splints to stack splint for acute mallet finger. Arch. Phys. Med. Rehabil..

[24] Pike J., Mulpuri K., Metzger M., Ng G., Wells N., Goetz T. (2010). Blinded, prospective, randomized clinical trial comparing volar, dorsal, and custom thermoplastic splinting in treatment of acute mallet finger. J. Hand Surg..

[25] Tocco S., Boccolari P., Landi A., Leonelli C., Mercanti C., Pogliacomi F., Sartini S., Zingarello L., Nedelec B. (2013). Effectiveness of cast immobilization in comparison to the gold-standard self-removal orthotic intervention for closed mallet fingers: A randomized clinical trial. J. Hand Ther. Off. J. Am. Soc. Hand Ther..

[26] Wilson S.W., Khoo C.T. (2001). The Mexican hat splint—A new splint for the treatment of closed mallet finger. J. Hand Surg..

